# Evidence for context-dependent functions of KDM5B in prostate development and prostate cancer

**DOI:** 10.18632/oncotarget.27818

**Published:** 2020-11-17

**Authors:** Bigang Liu, Rahul Kumar, Hseuh-Ping Chao, Rashid Mehmood, Yibing Ji, Amanda Tracz, Dean G. Tang

**Affiliations:** ^1^Department of Epigenetics and Molecular Carcinogenesis, The University of Texas M.D Anderson Cancer Center, Science Park, Smithville, TX, USA; ^2^Department of Pharmacology & Therapeutics, Roswell Park Comprehensive Cancer Center, Buffalo, NY, USA; ^3^Livestrong Cancer Institutes, Dell Medical School, The University of Texas at Austin, Austin, TX, USA; ^4^Department of Life Sciences, College of Science and General Studies, Alfaisal University, Takhasusi Street, Riyadh, Saudi Arabia; ^*^These authors contributed equally to this work

**Keywords:** KDM5B, prostate cancer, prostate development, hyperplasia, epigenetics

## Abstract

Prostate cancer (PCa) is one of the leading causes of cancer-related deaths worldwide. Prostate tumorigenesis and PCa progression involve numerous genetic as well as epigenetic perturbations. Histone modification represents a fundamental epigenetic mechanism that regulates diverse cellular processes, and H3K4 methylation, one such histone modification associated with active transcription, can be reversed by dedicated histone demethylase KDM5B (JARID1B). Abnormal expression and functions of KDM5B have been implicated in several cancer types including PCa. Consistently, our bioinformatics analysis reveals that the *KDM5B* mRNA levels are upregulated in PCa compared to benign prostate tissues, and correlate with increased tumor grade and poor patient survival, supporting an oncogenic function of KDM5B in PCa. Surprisingly, however, when we generated prostate-specific conditional *Kdm5b* knockout mice using probasin (Pb) promoter-driven *Cre: loxP* system, we observed that *Kdm5b* deletion did not affect normal prostate development but instead induced mild hyperplasia. These results suggest that KDM5B may possess context-dependent roles in normal prostate development vs. PCa development and progression.

## INTRODUCTION

The American Cancer Society estimates that > 190,000 new cases of prostate cancer (PCa) will be diagnosed in the United States in 2020 along with about 33,330 deaths [1]. The first-line therapy for PCa includes hormonal ablation or androgen deprivation therapy (ADT). Although ADT is effective in debulking tumors in most cases, the majority of treated PCa patients develop ADT resistance leading to emergence of more aggressive castration-resistant PCa (CRPC), which is the primary cause of PCa related deaths [2]. Development of acquired resistance to therapeutic regimens is common in virtually all targeted therapies and recent research implicates intratumor heterogeneity [3, 4]. Intratumor heterogeneity is maintained partly through the expression of distinct sets of genes among tumor cells, a phenomenon called cellular transcriptomic heterogeneity (CTH) [4], which is controlled by transcription factors and histone-modifying enzymes [3–6]. These enzymes work through regulating chromatin structure, which is an important determinant of gene activity. Chromatin, consisting of histones wrapped by DNA, is regulated by histone-modifying enzymes via methylation, phosphorylation, acetylation, ubiquitination, sumoylation, and ribosylation at lysine, arginine, serine, threonine, tyrosine, and other residues of histone tails [7, 8]. These histone modifications not only impact gene expression but also influence how the 3D structure of the chromatin is organized within the nucleus [9–12]. Depending on the type of modifications and the residues modified on the histone, the target gene can be transcriptionally activated or repressed. Genome-wide studies have indicated that histone acetylation is associated with higher transcriptional activity [5, 13, 14] whereas DNA methylation in the CpG islands is correlated with transcriptional repression. The methylation of histones impacts gene expression in a context-dependent manner. For example, trimethylation of histone 3 at lysine 4 and lysine 36 (H3K4me3 and H3K36me3) is generally associated with gene activation [15–18] whereas methylation at lysine 9 and 27 correlates with gene repression [16, 19–21].

KDM5B, also called JARID1B or PLU1, is a Jumonji C-containing (jmjC) histone lysine demethylase that plays important roles in organogenesis, stem cell functions and cancer development [6, 22]. KDM5B was initially identified as a critical regulator of embryonic cell differentiation (via decreasing H3K4 methylation in D3-D5 embryos) and as a determinant of zygotic genome activation and cellular fate changes during development [23]. In mouse embryonic stem cells, KDM5B binds various developmental genes to ensure proper neuronal differentiation [24]. Given its crucial role, *KDM5B* knockout in mice is embryonically lethal and the embryos exhibit extensive developmental defects [25]. These studies highlight an important role for KDM5B during normal development.

Genes involved in organogenesis and development are often dysregulated in tumorigenesis. Not surprisingly, KDM5B is overexpressed, and has been reported to play an oncogenic function, in a variety of cancers including breast cancer [4, 22], melanoma [26], PCa [27], lung cancer [28], hepatocellular carcinoma [29], gastric cancer [30], neuroblastoma [31] and leukemia [32]. On the other hand, potential tumor-suppressive functions of KDM5B have also been documented in some cases of melanoma and subtypes of breast cancer [33, 34]. Mechanistically, KDM5B has been reported to interact with transcription factors such as estrogen receptor α (ERα), androgen receptor (AR), progesterone receptor, PAX9, FOXG1, etc, and such interactions direct its localization to a diverse repertoire of genes in different cell types that further leads to distinct gene expression and contributes to CTH and intratumor heterogeneity [6].

Interestingly, KDM5B is specifically expressed in breast luminal cells and its loss induces basal-type gene expression, suggesting that KDM5B is a luminal lineage-driving oncogene in breast cancer [22]. Of note, KDM5B induces CTH and mediates therapeutic resistance in breast cancer [22]. As PCa is also a hormone-driven, luminal-type cancer with significant cellular heterogeneity and CTH [35, 36], KDM5B might play similar functions in PCa. Indeed, epigenetic events, mediated by histone-modifying enzymes, may be intimately involved in regulating AR signaling and PCa heterogeneity [27, 37, 38]. Our recent studies [35, 36] indicate that not only the expression of AR, but its transcriptional activity (as judged by the expression levels of AR transcriptional targets such as LRIG1) is also highly heterogeneous contributing to intratumor cellular heterogeneity in PCa.

Here, we first present bioinformatics data on KDM5B that supports an oncogenic function of KDM5B in PCa as previously reported [27]. We then present the surprising findings that *Kdm5b* deletion in the mouse prostate results in mild hyperplasia. Altogether, our study provides evidence for context-dependent roles of KDM5B in prostate organogenesis and tumorigenesis.

## RESULTS

### KDM5B mRNA is upregulated in PCa and correlates with genomic amplifications, tumor grade and poor survival

**Figure 1:**
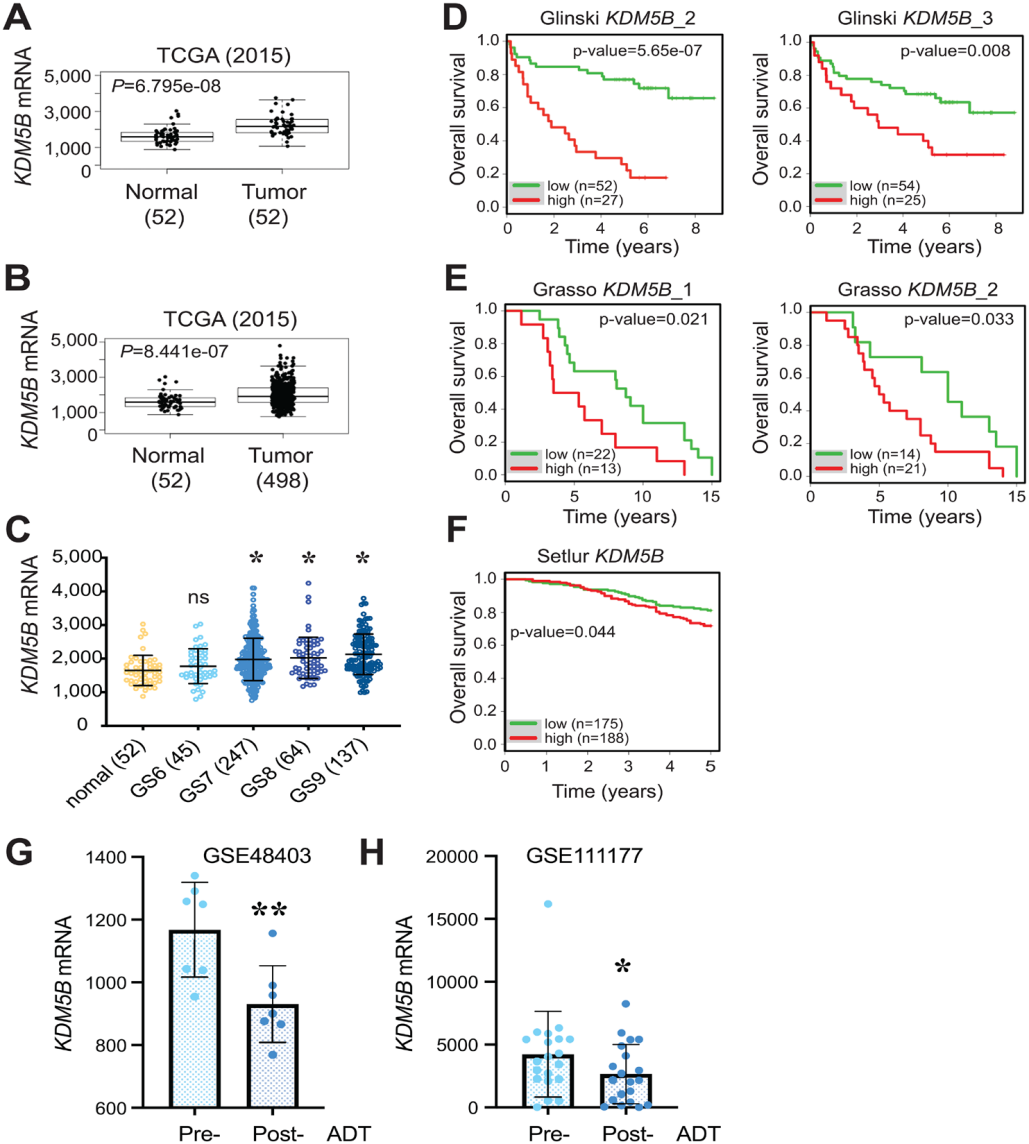
Overexpression of *KDM5B* mRNA in PCa and correlation with poor patient survival. (**A** and **B**) Elevated *KDM5B* mRNA levels in the TCGA PRAD dataset comparing 52 normal and 52 paired cancer tissues (A) or 52 normal and 498 cancer tissues (B). *P* values (two-tailed unpaired Student’s *t*-test) were indicated. (**C**) Elevated *KDM5B* mRNA levels in high-grade prostate tumors. Shown are *KDM5B* mRNA levels in the TCGA PRAD dataset PCa of increasing tumor grade (i.e., Gleason Score, GS6-10) and the 52 normal prostate tissues. ^*^ *P* < 0.05 compared with the normal (One-way ANOVA). ns, not significant. (**D**–**F**) High *KDM5B* mRNA levels correlate with poor PCa patient overall survival. Shown are Kaplan-Meier survival plots in the 3 indicated Oncomine datasets (1, 2, and 3 refer to different cDNA microarray probes used). *p*-values were determined using the Log-Rank test. (**G** and **H**) Reduced *KDM5B* mRNA levels in post-ADT patient tumors. KDM5B mRNA levels (read counts) were extracted from two RNA-seq datasets (GSE48403, *n* = 7; GSE111177, *n* = 20) and presented as the mean +/– S. D. ^*^ *P* = 0.032 and ^**^ *P* = 0.007 (two-tailed, paired Student’s *t*-test).

We first analyzed the *KDM5B* mRNA levels in GTEx (Genotype-Tissue Expression; https://gtexportal.org/) RNA-seq database. The results revealed wide expression of *KDM5B* mRNA across many human tissues including the prostate with the highest expression in the testis (Supplementary Figure 1A). Next we analyzed the *KDM5B* mRNA levels in TCGA PRAD dataset comparing normal and prostate tumor tissues. The *KDM5B* mRNA levels were significantly higher in PCa than the normal/benign tissue in both matched pair (52N/T; Figure 1A) and overall (52N/498T; Figure 1B) comparisons. Notably, *KDM5B* mRNA levels were elevated in PCa patient tumors with high tumor grade, i.e., combined Gleason Scores (GS) of 7–9 (Figure 1C). Furthermore, analysis of *KDM5B* mRNA levels in several Oncomine PCa datasets, including the Glinsky [39], Grasso (GSE35988; [40]) and Setlur (GSE8402; [41]) datasets revealed that high *KDM5B* mRNA levels correlated with poor PCa patients’ overall survival (Figure 1D–1F). These patients’ data, collectively, suggest an AR-regulated oncogenic role of KDM5B in PCa. Interestingly, when we interrogated the *KDM5B* mRNA levels in two RNA-seq datasets (GSE48403 and GSE111177) of matched patient tumors before ADT (pre-ADT) and post ADT failure (post-ADT), we observed reduced KDM5B mRNA levels in post-ADT tumors (Figure 1G and 1H). These results suggest that, consistent with an early study [27], KDM5B is regulated by AR.

An analysis of *KDM5B* mRNA levels in 31 human cancers in TCGA with the corresponding normal tissues pooled from TCGA and GTEx revealed increased or a trend of increased *KDM5B* mRNA in multiple cancers including bladder, breast, esophageal, pancreatic and prostate cancers as well as thymoma and acute myeloid lymphoma (Supplementary Figure 1B). Interestingly, *KDM5B*, most abundantly expressed in the normal testis (Supplementary Figure 1A), is reduced in testicular germ cell tumors (Supplementary Figure 1B). We further examined the mutational landscape of the *KDM5B* gene across a spectrum of cancers (Supplementary Figure 2). Multiple mutations representing amplification, missense mutations, and truncating mutations as well as copy number alterations (CNA) were observed in many cancers (Supplementary Figure 2A). Strikingly, in two hormone-driven cancers, breast (Supplementary Figure 2A) and prostate (Supplementary Figure 2B) cancers, we observed prevalent *KDM5B* genomic amplifications, which were accompanied by increased *KDM5B* mRNA expression in invasive breast cancer (BRCA; Supplementary Figure 1B), or increased (Figure 1A–1C) or an increased trend of (Supplementary Figure 1B) *KDM5B* mRNA levels in PCa. Several other cancers that showed genomic amplifications of *KDM5B* gene (Supplementary Figure 2A), e.g., thymic tumors (THYM), cholangiocarcinoma (CHOL), esophageal cancers (ESCA), GBM and bladder cancer (BLCA), also exhibited increased or trend of increased *KDM5B* expression (Supplementary Figure 1B). Overall, the analysis of genomic amplifications in *KDM5B* gene supports an oncogenic role of KDM5B in many human cancers including PCa. On the other hand, several cancers that manifested KDM5B genomic amplifications, e.g., hepatocellular carcinoma (LIHC) and ovarian cancer (OV), were not accompanied with (trend of) increased KDM5B mRNA expression (Supplementary Figure 1B; Supplementary Figure 2A).

### *Kdm5b* knockout induces mild hyperplasia in the mouse prostate

**Figure 2:**
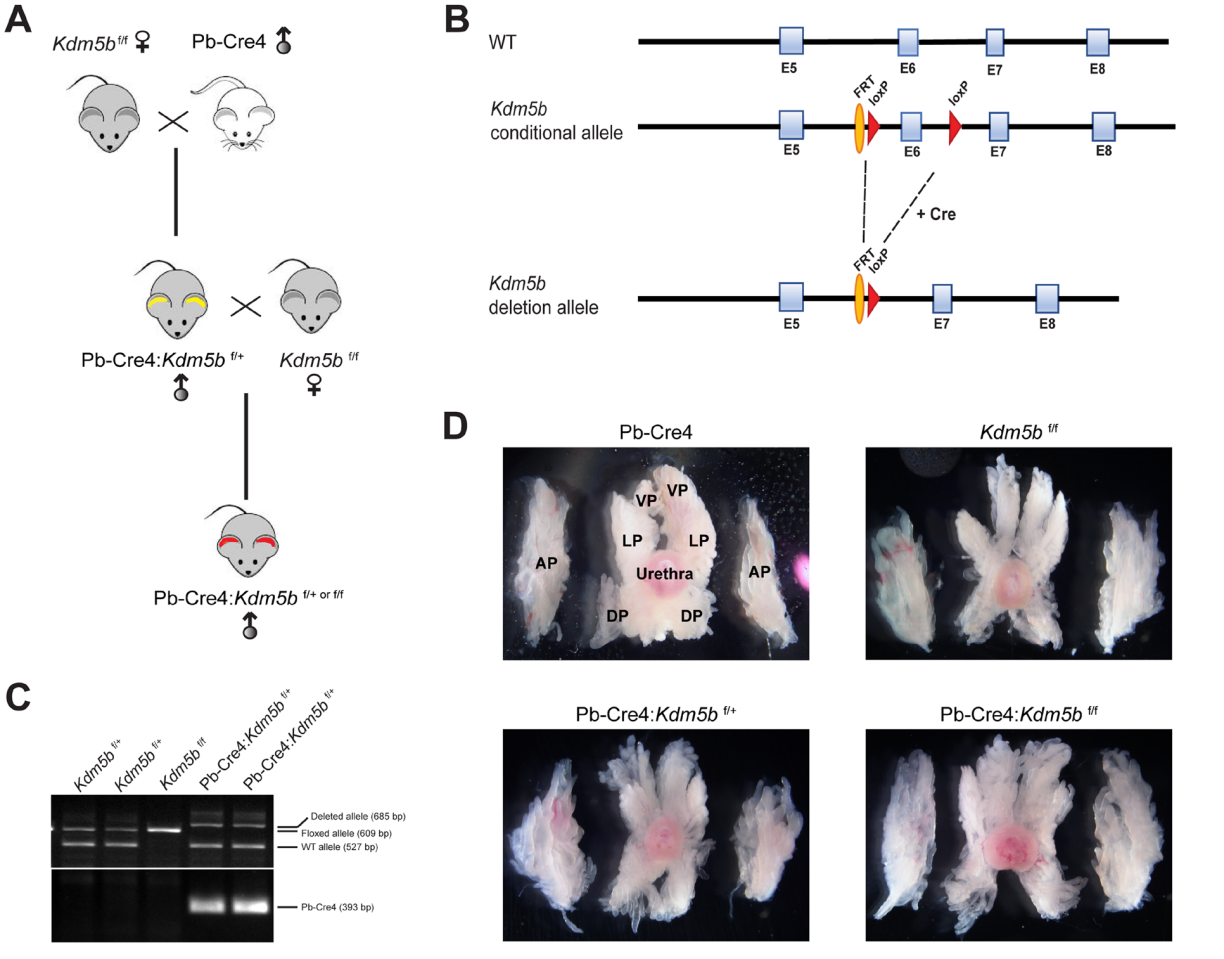
Generation of conditional *Kdm5b* knockout mice. (**A**) Breeding strategy for generating Pb-Cre4:*Kdm5b*^f/+^ or Pb-Cre4:*Kdm5b*^f/f^ mice. (**B**) Schematic of the *KDM5B* construct showing the loxP sites flanking the Exon (E) 6 of the *KDM5B* allele. (**C**) Representative genotyping gel image showing different *Kdm5b* alleles (Floxed, WT and deleted) and Pb-Cre4 PCR product bands. (**D**) Representative images of microdissected whole-mount prostates in WT and *Kdm5b*-deleted mice of different genotypes at 3 months of age (*n* = 3). AP, DP, LP and VP indicate anterior, dorsal, lateral, and ventral prostate lobes, respectively. The orientations of prostatic lobes are the same for all images shown.

Given the role of KDM5B in normal development and organogenesis (see Introduction), we intend to test its role in normal mouse prostate development in order to better understand its potential involvement in prostate tumorigenesis. To specifically knock out *Kdm5b* in the mouse prostatic epithelium, we crossed *Kdm5b-*floxed (*Kdm5b*^f/f^) mice [24] with the Pb-Cre4 line, which has Cre recombinase expression under the control of the ARR2PB promoter comprising a proximal element of the rat *Probasin* (Pb) promoter and two androgen responsive regions (Figure 2A and 2B). The ARR2PB promoter confines transgene expression predominantly in prostate luminal epithelial cells [42]. The mice were genotyped as described in the Methods with a representative gel image showing detection of the *Kdm5b* floxed and wilt-type (wt) alleles along with Pb-Cre4 (Figure 2C).

**Figure 3:**
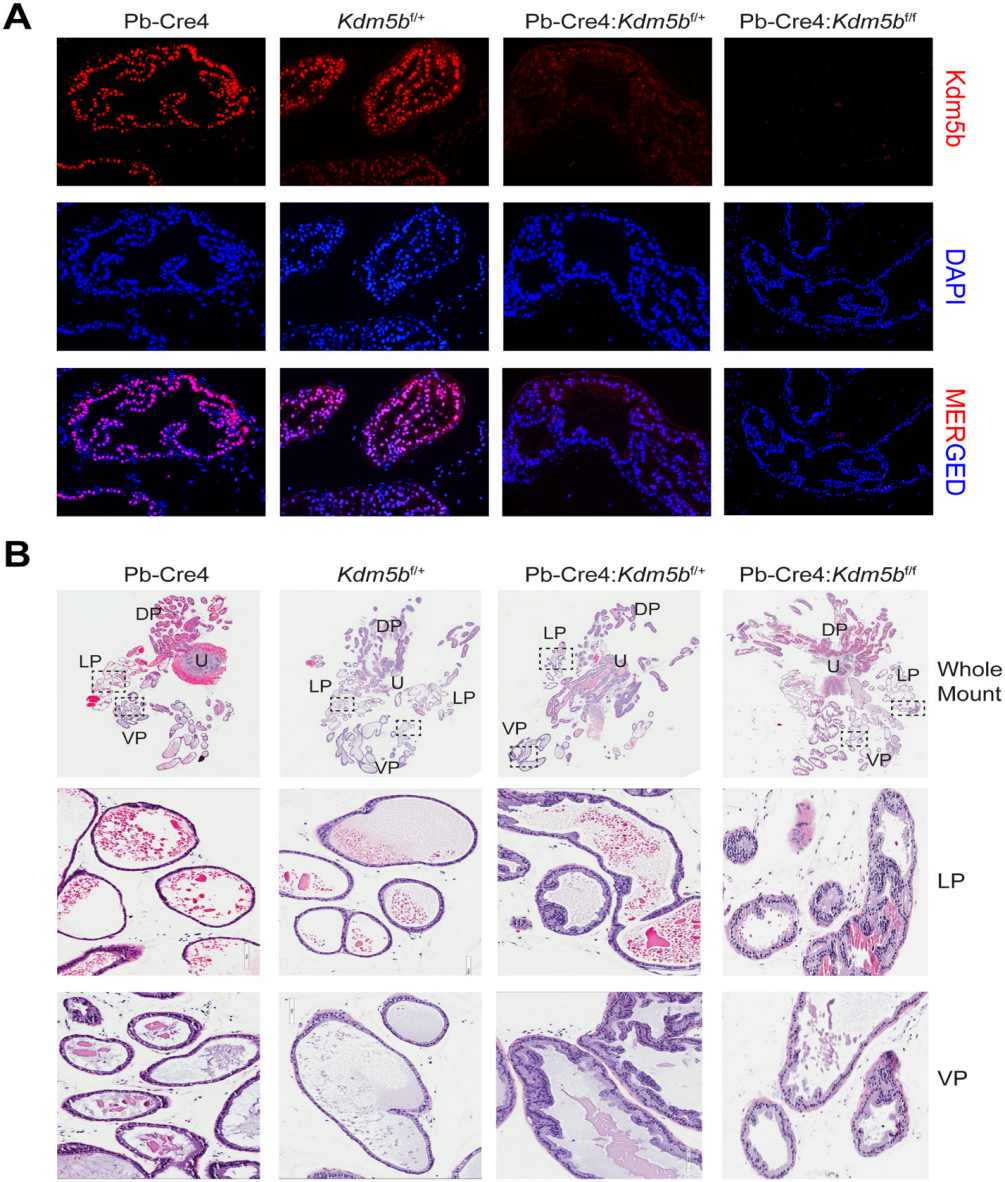
*Kdm5b* deficient mouse prostates manifest low-grade hyperplasia. (**A**) Immunofluorescence using anti-KDM5B antibody on formalin-fixed paraffin-embedded prostatic tissues obtained from the indicated genotypes. DAPI is used to stain the nuclei. (**B**) Representative HE images of whole-mount prostate sections (upper panels) from 3 months old mice of indicated genotypes. The middle and lower panel are representative blow-up images of LP and VP of the boxed areas in the whole mounts.

**Figure 4:**
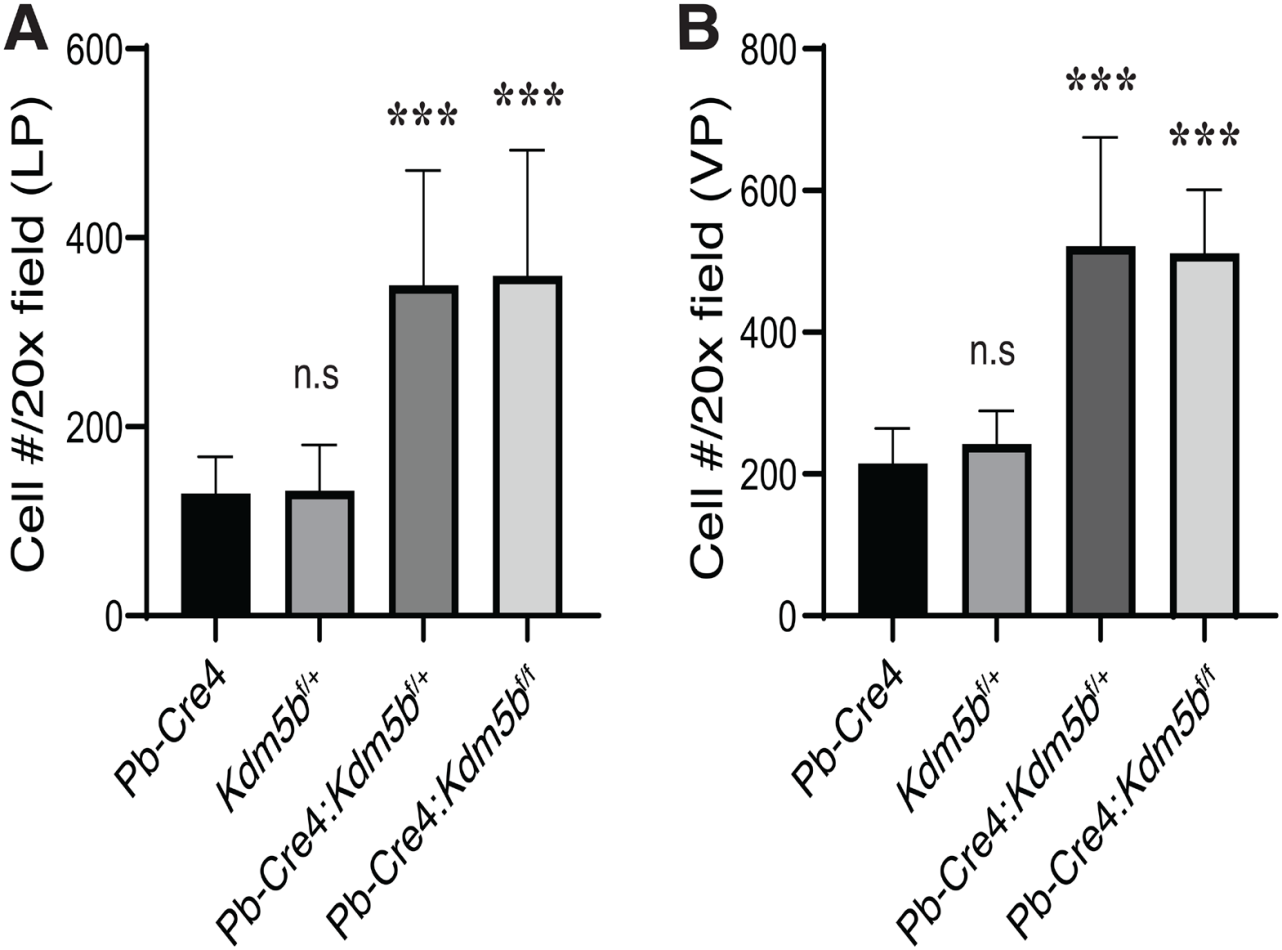
*Kdm5b* deficiency causes increased cellularity in the mouse prostates. The mouse LP (**A**) and VP (**B**) lobes were micro-dissected out from animals of the indicated genotypes (*n* = 8/genotype) as shown in Figure 3B. The number of cells, as identified by DAPI staining, were counted in 10 random microscopic fields under a 20× objective lens. Presented are bar graphs (mean +/– S. D) of the cell numbers per 20× field. ^***^ *P* < 0.0001 (Student’s *t*-test); n.s, not (statistically) significant.

*Kdm5b* knockout did not affect mouse prostate development (data not shown). Microdissected prostatic lobes (i.e., the anterior, dorsal, lateral and ventral prostates; AP, DP, LP and VP, respectively) from the 3-month-old mice of various genotypes showed overall similar gross morphologies and structures (Figure 2D). Immunofluorescence staining revealed beautiful and specific Kdm5b protein in the nucleus of luminal cells (Figure 3A). As expected, Kdm5b protein expression was significantly reduced in the heterozygous Pb-Cre4:*Kdm5b*^f/+^ mouse prostate and completely lost in the Pb-Cre4;*Kdm5b*^f/f^ prostate (Figure 3A). Interestingly, H&E analysis revealed mild hyperplasia in both heterozygous and homozygous *Kdm5b*-knockout mouse prostate, especially in the LP and VP (Figure 3B). In the control (i.e., Pb-Cre4 and *Kdm5b*^f/+^) mice, the LP and VP consisted of uniform glands with single luminal epithelial cell layer (Figure 3B). In contrast, the *Kdm5b*-deleted hyperplastic glands displayed thickened luminal cell compartment with more than one cell layer and, frequently, papillary structures protruded into the lumen (Figure 3B). In support of the hyperplasia phenotype, quantitative analysis revealed significantly increased cellularity in both the LP and VP of Pb-Cre4:*Kdm5b*^f/+^ and Pb-Cre4:*Kdm5b*^f/f^ prostates (Figure 4A and 4B). Hyperplasia persisted, but no PIN (Prostate Intraepithelial Neoplasia) or apparent tumors developed, in 1–1.5 years old Pb-Cre4:*Kdm5b*^f/+^ and Pb-Cre4:*Kdm5b*^f/f^ prostates (data not shown).

## DISCUSSION

Epigenetic modifications lie at the heart of normal development and organogenesis, and analysis of epigenetic landscape comparing various epigenetic modifications between normal and cancer tissues has revealed massive epigenetic dysregulation during cancer development. Molecular dissection of the epigenetic modifiers may shed light on their roles in normal vs. cancer development. In the present study, we provide evidence that KDM5B, a H3K4 demethylase, may exhibit two contrasting functions: in human PCa, it is significantly upregulated (consistent with an earlier report; 27) and correlates with poor patient survival thus pointing to an oncogenic role; in contrast, genetic deletion of *Kdm5b* leads to mild hyperplasia in the mouse prostate, pointing to a potentially tumor-suppressive function.

KDM5B has been traditionally thought to repress transcription since it catalyzes the demethylation of H3K4me1/me2/me3: H3K4me2/me3 are enriched at the promoter region of actively transcribed genes [16] while H3K4me1 marks the enhancer regions [43–45]. Surprisingly, KDM5B is overexpressed (or shows the trend of overexpression) in many human cancers in addition to PCa (Supplementary Figure 1B). Though the role of KDM5B in tumorigenesis is not well understood, its higher expression in cancer cells may regulate the distribution of H3K4me3 near promoter regions of tumor suppressors and modulate their expression, thus affecting cancer cell proliferation [6]. Recently, several KDM5B inhibitors have been identified and reported in different cancers such as the breast and prostate cancers [46, 47]. In all these studies, genetic or pharmacologic inhibition of KDM5B upregulates the expression of tumor suppressor genes and caused growth arrest and apoptotic cell death in cancer cells suggesting that KDM5B may mostly act as a repressor of tumor suppressor genes. KDM5B inhibitors have also been reported to overcome radioresistance in cancer cells by preventing the demethylation of H3K4 at the sites of double-strand breaks induced by radiation [48]. Therefore, DNA damage repair machinery failed to resolve the damage leading to increased radiosensitivity of KDM5B-overexpressing cancer cells. Overall these studies suggest a tumor promoting potential of KDM5B, which is consistent with our observations that it is highly expressed in human PCa and its expression correlates with poor patient survival.

Interestingly, *Kdm5b* deletion in the mouse prostate leads to mild hyperplasia, which is surprising given its reported pro-oncogenic role in prostate and other cancers. Earlier studies demonstrate that KDM5B is not only overexpressed in hormone-driven cancers such as the breast and prostate cancers, but also interacts with hormone receptors, ER and AR, to positively regulate their transcriptional activities [25, 27]. Therefore, it is plausible to speculate that KDM5B might function in the prostate and PCa through modulating AR signaling. In partial support of this connection, KDM5B mRNA levels were significantly reduced in post-ADT (castration-resistant) patient prostate tumors (Figure 1G and 1H). Whether the hyperplasia in *Kdm5b*-deficient prostate could be related to anomalies in the AR signaling axis is currently under investigation. On the other hand, increased cellularity in *Kdm5b*-deleted mouse prostate (Figure 3B and Figure 4) implies that Kdm5b might demethylate the H3K4 in the promoter/enhancer regions of tumor-promoting genes and keep them in a repressed state during normal prostate development. *Kdm5b* deletion in the prostate leads to upregulation of these genes and subsequent luminal hyperplasia. Further experiments such as RNA-seq analysis of the *Kdm5b*-depleted mouse prostate need to be performed to reveal the full spectrum of its functions in prostate development.

Collectively, our data suggest that KDM5B may possess context-dependent functions in prostate organ development and prostate tumorigenesis. As it has been previously reported that KDM5B interacts with a variety of transcription factors and such interactions determine its localization on the specific set of genes, it will be very interesting to investigate its interacting partners during different stages of prostate and PCa development.

## MATERIALS AND METHODS

### KDM5B bioinformatics, survival and mutational landscape analyses

General strategies in bioinformatically analyzing KDM5B mRNA expression and correlation with patient survival have been described in our recent publications [35, 36]. Briefly, *KDM5B* mRNA levels in normal human tissues (Supplementary Figure 1A) were extracted from the GTEx (Genotype-Tissue Expression) data portal (https://gtexportal.org/). For TCGA data analysis (Figure 1A–1C), we obtained TCGA level-3 data for *KDM5B* mRNA from TCGA data portal (https://tcga-data.nci.nih.gov). We performed the Student’s *t*-test for normal and tumor tissue comparisons and one-way ANOVA for determining expression levels among different Gleason scores, respectively (Figure 1A–1C).

We performed survival analysis and generated Kaplan-Meier survival plots using the survival package in R (Figure 1D–1F). In brief, we obtained the individual normalized gene expression data from patients with both survival time and survival status from Oncomine (https://www.oncomine.com; Compendia Bioscience) datasets and ranked the data according to *KDM5B* mRNA expression. Then, we assigned the sample with rank from the first quartile to the third quartile into two groups and compared the *p*-value between these two groups along with different cutoffs. Finally, we set the ultimate cutoff with the smallest *p*-value and plotted a Kaplan-Meier survival curve.

We also compared *KDM5B* mRNA expression levels of 31 human tumors in TCGA with the corresponding matched normal tissues pooled from TCGA and GTEx (Supplementary Figure 1B). Briefly, KDM5B gene expression across the 31 cancer types and paired normal samples was generated from GEPIA (http://gepia.cancer-pku.cn), with each dot representing a distinct tumor (from TCGA) or normal sample (from TCGA and GTEx). Four-way analysis of variance (ANOVA) was employed, using sex, age, ethnicity and disease state (Tumor or Normal) as variables, to determine differential expression. The expression data are first log_2_(TPM+1) transformed and the log_2_FC is defined as median (Tumor) - median (Normal). The Benjamini and Hochberg false discovery rate (FDR) method was used to adjust.

Finally, we analyzed the *KDM5B* mutations in the prostate and multiple cancer datasets using cBioportal (http://www.cbioportal.org) (Supplementary Figure 2).

### Generation of conditional KDM5B knockout mice

Generation of conditional knockout mice with a floxed *Kdm5b* allele has been described previously [24]. The overall breeding strategy is illustrated in Figure 2A. Male PB-Cre4 mice were crossed with female *Kdm5b*^f/f^ to obtain PB-Cre4:*Kdm5b*^f/+^ or PB-Cre4:*Kdm5b*^f/f^ mice. For genotyping, genomic DNA was isolated from tail snips using EZNA tissue DNA kit (Omega Bio-Tek, GA, USA) as per manufacturer’s instructions. Briefly, tail snips were minced in supplied TL buffer and Proteinase K solution followed by incubation at 55°C overnight. Genomic DNA were purified using supplied HiBind DNA mini columns and 2 μl of genomic DNA extracts were subjected to PCR reaction for genotyping of different alleles (WT and floxed; PB-Cre4) using the following primers: *Kdm5b floxed allele* (Fwd: CCCTGG-GATTGCAGTTAAAG; Rev (floxed allele): TGGCTTCCACAATCTTCAATG; Rev (deleted allele): GTCAACTGCAAACTGACCTCTG; PCR product size: WT = 527 bp; floxed allele = 609 bp; and deleted allele = 685 bp), and Pb-Cre4 transgene (Fwd: CTGAAGAATGGGACAGGCATTG; Rev: CATCACTC-GTTGCATCGACC; PCR product size: 393 bp).

### Prostate isolation, microdissection, Aperio ScanScope analysis, and immunofluorescence

Basic procedures for these experiments have been previously described [35, 36, 49–52]. After sacrificing mice at the age of 3 months, the prostates were surgically removed along with the urogenital tract. The prostates were placed immediately in ice-cold phosphate-buffered saline (PBS) and microdissected under a dissection microscope to remove fat and connective tissues. The isolated whole-mount prostates were photographed with a Nikon digital camera and then fixed in 10% formalin for further histological analysis. For Aperio Scanscope analysis, H&E-stained glass slides containing sections of WM mouse prostates were scanned via an Aperio ScanScope imaging platform (Aperio Technologies, Vista, CA, USA) and images analyzed on Imagescope analysis software.

For immunofluorescence staining, formalin fixed mouse prostate sections were deparaffinized using xylene and rehydrated through incubating sections in serial decreasing concentrations of ethanol (95–50%). After washing with distilled water, antigen was retrieved in antigen-retrieval solution (Sodium citrate buffer, pH6.0). After washing with wash buffer (Tris Buffered Saline-Tween 20), sections were blocked with Background sniper (Biocare Medical, CA, USA) for 1 hr at room temperature (RT) followed by anti-KDM5B antibody (Bethyl lab, Cat # A301-813A) incubation at 4°C for overnight. After washing with wash buffer, sections were incubated with anti-rabbit IgG-PE secondary antibody (Santa Cruz, Cat # sc-3753) for 1 hr at RT followed by 10 minutes incubation in DAPI (0.1 μg/ml). Sections were washed extensively with wash buffer and mounted with Prolong Gold mounting medium (Thermo Fisher Scientific, MA, USA).

### Statistical analysis

Statistical analyses were performed using GraphPad Prism software or using R. In general, paired or unpaired two-tailed Student’s *t*-test was used to calculate the statistical significance between comparisons. Differences in patients’ overall survival were determined by the Log-Rank Test. *P* < 0.05 is considered statistically significant.

### Ethics statement

All animal work was approved by our institutional animal care and use committee at the University of Texas M.D Anderson Cancer Center or the Roswell Park Comprehensive Cancer Center. All animals were maintained in standard conditions according to the institutional guidelines. Animal housing rooms were under temperature and humidity control and mice were not subjected to water or food restrictions.

## SUPPLEMENTARY MATERIALS


