# Supplementary material for: Evidence for context-dependent functions of KDM5B in prostate development and prostate cancer
Source: Oncotarget. 2020 Nov 17;11(46):4243–52. doi: 10.18632/oncotarget.27818 (PMC7679033; doi:10.18632/oncotarget.27818)
Supplement: Supplementary file 1 [file oncotarget-11-4243-s001.pdf]

Evidence for context-dependent functions of KDM5B in prostate development and prostate cancer

SUPPLEMENTARY MATERIALS

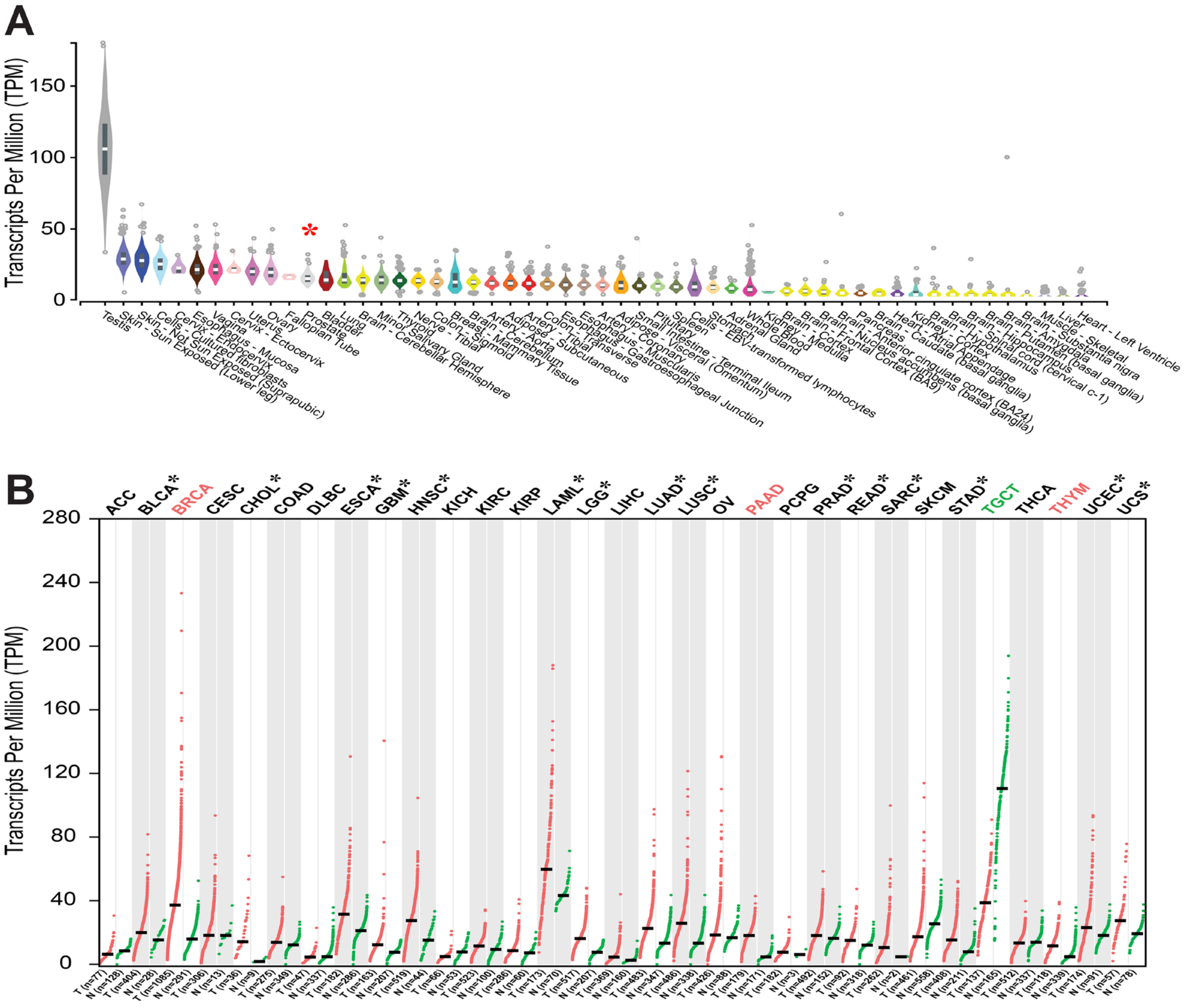

Supplementary Figure 1: *KDM5B* mRNA expression in normal human tissues (**A**) and in human cancers compared to the normal tissues (**B**). (A). *KDM5B* mRNA levels (TPM; Transcript per Million) in more than 50 human tissues (or cells) retrieved from GTEx (ENSG00000117139.16). The prostate is indicated with a red asterisk. (B). KDM5B mRNA expression in 31 human cancers compared to matched normal tissue (see Methods). The *KDM5B* mRNA levels (TPM) in tumors (from TCGA) and matched normal tissues (from both TCGA and GTEx) are upregulated in 3 (red) and downregulated in 1 (green) human cancers (indicated above; $P < 0.05$, 4-way ANOVA). The abbreviations for each cancer are shown below. Note that 15 tumors, indicated by *, showed a trend of increased KDM5B mRNA in tumors. ACC: Adrenocortical carcinoma; BLCA: Bladder Urothelial Carcinoma; BRCA: Breast invasive carcinoma; CESC: Cervical squamous cell carcinoma and endocervical adenocarcinoma; CHOL: Cholangiocarcinoma; COAD: Colon adenocarcinoma; DLBC: Lymphoid Neoplasm Diffuse Large B-cell Lymphoma; ESCA: Esophageal carcinoma; GBM: Glioblastoma multiforme; HNSC: Head and Neck squamous cell carcinoma; KICH: Kidney Chromophobe; KIRC: Kidney renal clear cell carcinoma; KIRP: Kidney renal papillary cell carcinoma; LAML: Acute Myeloid Leukemia; LGG: Brain Lower Grade Glioma; LIHC: Liver hepatocellular carcinoma; LUAD: Lung adenocarcinoma; LUSC: Lung squamous cell carcinoma; OV: Ovarian serous cystadenocarcinoma; PAAD: Pancreatic adenocarcinoma; PCPG: Pheochromocytoma and Paraganglioma; PRAD: Prostate adenocarcinoma; READ: Rectum adenocarcinoma; SARC: Sarcoma; SKCM: Skin Cutaneous Melanoma; STAD: Stomach adenocarcinoma; TGCT: Testicular Germ Cell Tumors; THCA: Thyroid carcinoma; THYM: Thymoma; UCEC: Uterine Corpus Endometrial Carcinoma; UCS: Uterine Carcinosarcoma.

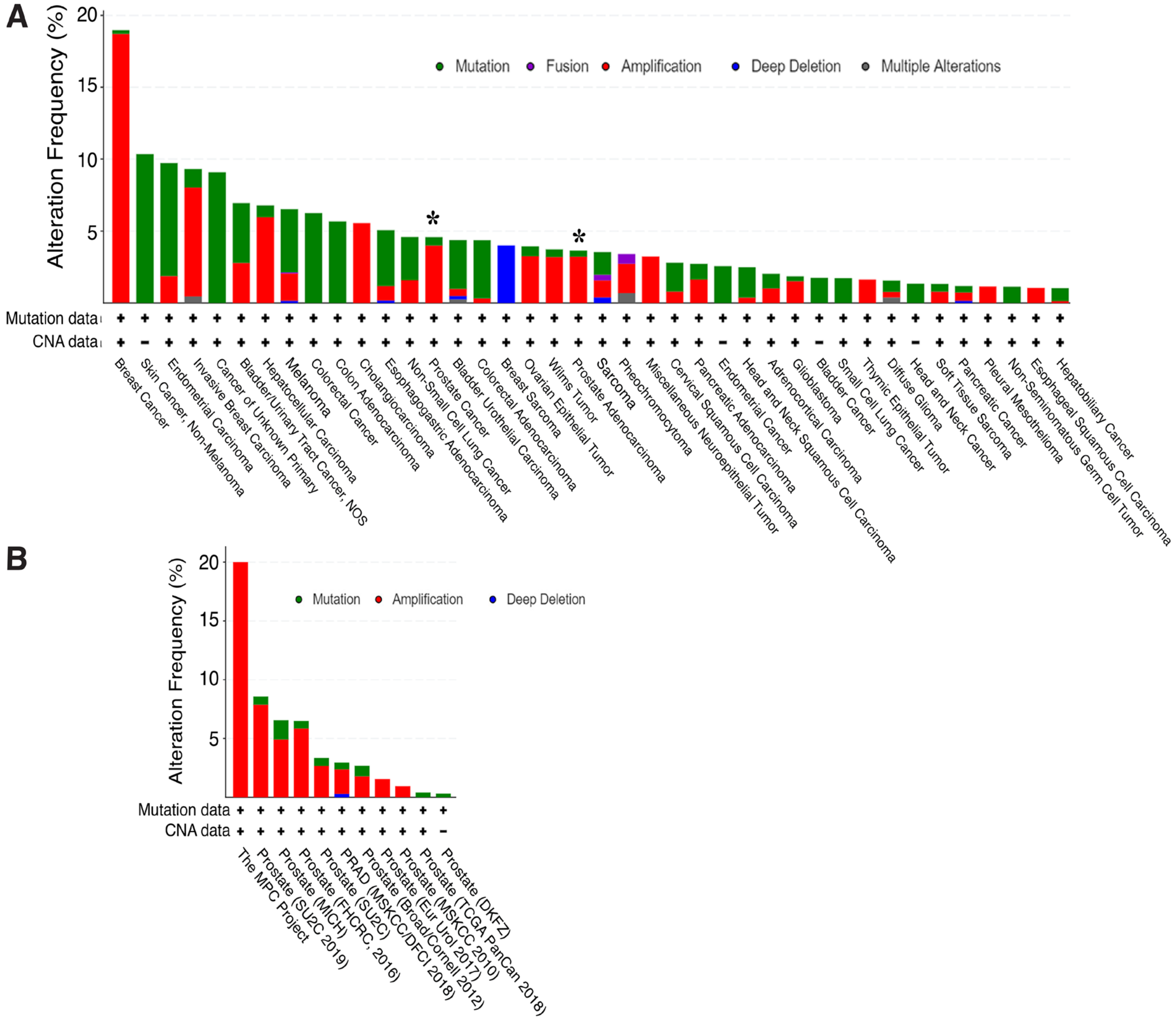

Supplementary Figure 2: Mutational landscape of the KDM5B gene in human cancers. cBioportal datasets have been analyzed for mutational landscape of KDM5B in pan-cancer types (**A**) and PCa (**B**). In (A), the two PCa datasets were indicated by asterisks. In (B), 11 PCa datasets (indicated in parentheses) were analyzed.
